# An epidemic of mass hysteria in a village in West Bengal

**DOI:** 10.4103/0019-5545.55956

**Published:** 2005

**Authors:** A.N. Chowdhury, A. Brahma

**Affiliations:** * Professor and Head, Institute of Psychiatry, 7 D.L. Khan Road, Kolkata 700025; ** DNB (Psychiatry) Trainee, Institute of Psychiatry, 7 D.L. Khan Road, Kolkata 700025

**Keywords:** Mass hysteria, psychiatric epidemic

## Abstract

This is a report of an outbreak of mass hysteria, attributed to an unknown infectious disease, in a small village near Baruipur, South 24 Parganas district of West Bengal. The epidemic was triggered following the death of 2 persons of the same family on the same day. Thereafter, several other family members and villagers became ill and complained of similar symptoms. They were hospitalized for observation and all were discharged after a couple of days. We report the detailed sequence of events and the management of this mini epidemic.

## INTRODUCTION

Mass hysteria, also known as mass psychogenic illness (MPI), has been reported for hundreds of years in different sociocultural settings. MPI has been characterized by a group of symptoms that usually mimic an organic disease, but without any identified cause, and occurs in those who share a common belief that those symptoms constitute a definite illness.^1^ Boss^2^ considered this to be a social phenomenon that affects otherwise healthy individuals. The outbreaks often have an environmental trigger such as odours,^3^ water or food.^4^,^5^ Involvement of young people with a preponderance of females is reported in the literature.^2^ Outbreaks are often characterized by the rapid spread of symptoms by ‘line of sight trans-mission’, i.e. directly watching other affected persons.^3^ The role of the media is also important as a positive reinforcer of MPI,^6^ which was evident in the epidemic of mass hysteria concerning an imaginary threat from a ‘monkey man’ in East Delhi.^7^

## THE EPIDEMIC

An outbreak of mass hysteria attributed to an infectious disease in Ganga Duara, a village near Baruipur, occurred on Monday, 14 July 2003. A local news daily reported the mass illness behaviour frenzy throughout the village as follows:

A dreaded situation occurred in Ganga Duara village of Baruipur following two deaths due to jaundice in the same family. Panchu Naskar and his daughter Anjana died of jaundice on Sunday. In the same family 8 other members became ill along with one member of the neighbouring house and all of them were admitted at Baruipur rural hospital on Monday. On the other hand, big excitement was noticed on Monday in Ganga Duara village after hearing the news of 2 deaths and 9 others admitted in hospital because of serious illness. Today the superintendent of National Medical College and Hospital, Kolkata told that they died of hepatic encephalopathy…One medical officer of Baruipur hospital opined that primarily it was suspected that all these 9 cases became ill as they became frightened following the death of Panchu and Anjana…^8^

Apparently, the first symptom of this infectious disease began in the early morning of 14 July 2003 and, within a few hours, it spread to the whole family and then the whole village. Both Panchu Naskar and his daughter Anjana had been sick for the past 15 days but no medical help had been sought. On 13 July 2003, both of them were brought to the Baruipur hospital with deep jaundice. They were referred immediately to the Calcutta National Medical College and Hospital as both were comatose at that time. Anjana had a serum bilirubin level higher than 10 and was negative for the hepatitis B surface antigen. Her father did not have any laboratory investigation report. Unfortunately, both of them died on the same day—Anjana in the afternoon and Panchu at midnight. The attending physician suspected that both of them had died of fulminant hepatic encephalopathy.

The family was shocked at the news of the two deaths. The index case was Pintu, the 19-year-old son of Panchu, who started crying in grief. Suddenly, he experienced a tingling sensation over his limbs with loss of control, along with extreme uneasiness and yellowish discoloration of the palms. The other family members rushed to help him. Mrs Rekha Bayen, the wife of the Gram Panchyat's *Pradhan,* also came along with them as she was their neighbour and had a good relationship with the Naskar family. She suggested that Pintu be sent immediately to a hospital because she was convinced that Pintu had a serious illness. She narrated her own experience regarding a similar peculiar sensation a few years ago for which she was treated at a hospital. She also stated that it might be an infectious disease that had caused the death of his father and sister. Her experience and suggestion were seriously taken and accepted.

Immediately, Pintu was taken to a local nursing home. Soon after, several other family members developed similar symptoms. Within a few hours, people of different age groups were affected and the victims were taken to the local hospital by van-rickshaw. Some of them complained of electric shock-like sensation over their legs along with paraesthesia over the body. Sanjay, Panchu's son-in-law, was very anxious that it might be an infectious disease, which was spreading rapidly and might cause death. So he jumped into a roadside pond in the belief that the germs might wash off from his body.

In the meanwhile, fear of a dreaded infectious disease spread throughout the village. The intensity of apprehension about the ‘mysterious infectious disease’ was so severe that there was a procession of members of the Naskar family awaiting admission to hospital. A total of 11 persons were admitted to the local hospital from that village within a time span of 9 hours. The atmosphere in the entire village was tense. Villagers started jumping into ponds to remove the dreaded ‘infectious germ’ from their bodies. Intense media coverage quickly reported widespread illness due to an unknown infectious agent. The next day, two other members of the Naskar family were admitted to the hospital with the same complaints. Interestingly, two patients who were admitted in that hospital for some other complaints, also developed symptoms of faint yellowish discoloration of the palms and soles, and tingling of the limbs after observing members of the Naskar family.

In the hospital, the patients were examined thoroughly and investigations were done. No organic or laboratory abnormalities were found that would explain the symptoms. Fifteen people were admitted for observation and all were discharged after a couple of days; they did not have any complication. A medical team consisting of an epidemiologist, psychiatrist and public health specialist went to the village. A meeting was arranged with the help of the local Panchayat and villagers were assured that it was not an infectious disease which caused the death of two members of the Naskar family. They were told about hepatic encephalopathy, which was the cause of death in these two. The cases subsequently admitted in hospital did not have such a disease; there was no jaundice or any other symptom. The bereaved family members were so anxious and perplexed that out of their fear, they not only developed similar symptoms but also helped to spread the fear of ‘mysterious infective disease’ among others. Villagers were convinced about this logic and started community canvassing against this fear. There was no further case.

Table 1 shows that following the two deaths, a total of 11 subjects were affected on 14 July 2003 within a time interval of 9 hours. Of them, 10 were from the same family. On the next day, i.e. 15 July 2003, 2 more persons from the Naskar family became ill and were hospitalized. Two subjects already admitted in hospital for some other complaints, also developed similar symptoms.

**Table 1:** Time sequence and sex-wise distribution of the cases

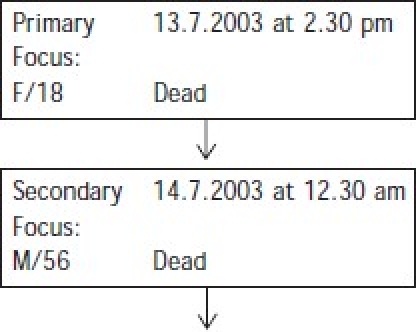				
Age (in years)	Sex	Time of onset of disease	Date of onset of disease	Relationship
19	M	6.00 am	14.07.03	Index case (member of the Naskar family)
14	M	7.00 am	14.07.03	Member of the Naskar family
18	M	7.30 am	14.07.03	"
30	M	8.30 am	14.07.03	"
54	M	8.30 am	14.07.03	"
21	F	8.30 am	14.07.03	"
35	F	11.30 am	14.07.03	Member of other family
16	M	12.50 pm	14.07.03	Member of the Naskar family
35	F	1.30 pm	14.07.03	"
17	F	2.20 pm	14.07.03	"
22	F	3.00 pm	14.07.03	"
36	F	7.00 am	15.07.03	"
25	F	8.00 am	15.07.03	"
24	F	9.00 am	15.07.03	Case from within the hospital
28	F	10.00 am	15.07.03	"

Table 2 shows that the most frequent symptoms were yellowish discoloration of the palms and soles (93.3%). It is interesting to note that both the deceased manifested this symptom before their death.

**Table 2:** Symptom profile of the cases

	Number of cases		
	
Symptom	Male (*n*=6)	Female (*n*=9)	Total (%) *n*=15
Tingling sensation over the limbs	4	8	12 (80)
Electric shock-like sensation over the legs	2	1	3 (20)
Yellowish discoloration of the palms and soles	6	8	14 (93.3)
Paraesthesia over the body	3	7	10 (66.7)
Headache	4	8	12 (80)
Eye pain	2	6	8 (53.3)
Weakness	5	7	12 (80)
Difficulty in respiration	2	3	5 (33.3)
Burning sensation on the scalp	—	4	4 (26.7)
Difficulty in swallowing	—	1	1 (6.7)

## DISCUSSION

This mini MPI is an excellent example of how a mass hysterical response was triggered by the tormenting anxiety following the sudden death of two family members. Detailed field enquiry revealed that the first clue to ‘symptoms’ of an ‘infectious disease’ came from a woman who had a Koro attack (breast symptoms only) 18 years back. Her positive suggestion of an alleged illness favoured the hysterical symptom choice among the bereaved and anxious family members of the deceased. Once the symptoms were expressed and socially accepted as an illness paradigm, others were affected in a chain reaction. Anxiety, ignorance about the illnesses of the deceased, knowledge of the familial relationship, vulnerability to the spread of the disease and the conducive cultural atmosphere helped the outbreak of a mass psychogenic reaction.^9^ Mattoo *et al.*^10^ in their study of mass family hysteria in an extended family showed how 10 out of 31 family members were affected in a background of strong religious and cultural beliefs of the closely knit family kinship system. False beliefs about a hypothetical illness always play an important role in the spread of the illness–contamination fear as evidenced in the study by Dhar *et al.*^11^ in which alleged food poisoning triggered a mass hysterical response in the community.

Intervention should be as quick as possible. In this case, immediate steps were taken and meetings with the villagers helped to check further spread of the illness in the village. Proper information to the affected persons as well as the community,^12^ public health education and training of primary care physicians may help to check the spread of such mass hysterical episodes^13^ in the community.
